# Antidepressant utilization after hospitalization with depression: a comparison between non-Western immigrants and Danish-born residents

**DOI:** 10.1186/1471-244X-14-77

**Published:** 2014-03-17

**Authors:** Helle Wallach-Kildemoes, Louise Thirstrup Thomsen, Margit Kriegbaum, Jørgen Holm Petersen, Marie Norredam

**Affiliations:** 1 Centre for Healthy Ageing, Faculty of Health Sciences, University of Copenhagen, Copenhagen, Denmark; 2 Institute of Pharmacy, Faculty of Health Sciences, University of Copenhagen, Copenhagen, Denmark; 3 Danish Research Centre for Migration, Ethnicity and Health, Section for Health Services Research, Department of Public Health, Faculty of Health Sciences, University of Copenhagen, Copenhagen, Denmark; 4 Section for Biostatistics, Department of Public Health, Faculty of Health Sciences, University of Copenhagen, Copenhagen, Denmark; 5 Section of Immigrant Medicine, Department of Infectious Diseases, Copenhagen University Hospital, Hvidovre, Denmark

**Keywords:** Immigrants, Post-remission, Antidepressant, Ethnic disparities, Health care

## Abstract

**Background:**

Antidepressant (AD) therapy is recommended for patients 4–12 months after remission from depression. The aim was to examine whether immigrants (refugees or family reunited immigrants) from non-Western countries are at greater risk than Danish-born residents of 1) not initiating AD therapy after discharge and 2) early AD discontinuation.

**Methods:**

A cohort of immigrants from non-Western countries (n = 132) and matched Danish-born residents (n = 396) discharged after first admission with moderate to severe depression between 1 January 1996 and 31 May 2008 was followed in the Danish registries.

Logistic regression models were applied to explore AD initiation within 30 days after discharge, estimating odds ratio (OR) for immigrants versus Danish-born residents.

Early discontinuation was explored by logistic regression, estimating OR for no AD dispensing within 180 days after the first dispensing, and by Cox regression, estimating hazard ratio (HR) for discontinuation (maximum drug supply gap) within 180 days.

**Results:**

Immigrants had higher odds for not initiating AD treatment after discharge than Danish-born residents (OR = 1.55; 95% CI: 1.01-2.38). When income was included in the model, the strength of the association was attenuated. Odds for early discontinuation was non-significantly higher among immigrants than Danish-born residents (OR = 1.80; 0.87-3.73). Immigrants also had a non-significantly higher hazard of early discontinuation (HR = 1.46; 95% CI: 0.87-2.45). Including income had only minor impact on these associations.

**Conclusion:**

Immigrants seem less likely to receive the recommended AD treatment after hospitalization with depression. This may indicate a need for a better understanding of the circumstances of this vulnerable group.

## Background

The number of immigrants in Denmark has grown over the last 30 years. As of 1 January 2012, immigrants (7.9%) and their descendants (2.5%) constituted 10.4% (580,461) of the population [1]. Several European studies have found an increased prevalence of self-reported depression [2,3] and/or psychological distress [4] in immigrant groups compared with local born European populations, and it has been hypothesized that loss and trauma associated with immigration may increase the risk of depression in vulnerable individuals [5]. In Denmark, the most frequent countries of origin (excluding the European Union) for immigrants are: Turkey, Iraq, Bosnia, Iran, Lebanon, Pakistan, Afghanistan, and the former Yugoslavia [1].

In Denmark, access to health care service is formally independent of migrant status, owing to a health care system based on principles of free and universal access for all residents with equal treatment for equal needs [6]. Co-payment is, however, required for important services such as psychotherapy and prescription drugs [7].

Studies from both Denmark [8] and other countries [9,10] indicate that immigrants or ethnic minority groups may not receive care of the same quality as majority populations explained for example by communication difficulties between health care staff and minority patients [11-13]. From an immigrant health perspective, it is therefore important to examine the quality of care for immigrants with depression.

For patients with moderate to severe depression, current guidelines recommend pharmacological treatment for at least 4–12 months after remission to reduce the risk of relapse [14-17]. However, studies indicate that 20–70% of patients discontinue treatment prematurely [18-22]. Dutch, Spanish, and American studies have found that immigrants and/or ethnic minority patients with depression have lower odds of initiating antidepressant (AD) treatment [23,24] and a higher risk of early treatment discontinuation [9,10,21,25,26] than the majority population. Other studies have found ethnic differences in patients’ beliefs and attitudes towards psychopharmacological treatment [27,28] and ADs [29], which may influence patient medication taking behavior [30-32]. Furthermore, a Danish register-based study among patients treated with ADs in general practice [19] found that patients with low income or low educational level had a higher risk of discontinuing treatment within six months than patients with higher socio-economic position (SEP). This study also found that foreign citizens had a twofold higher risk than Danish citizens of discontinuing AD treatment within six months [19], but this finding was not further explored or discussed by the authors. Since non-Western immigrants in Denmark on average have lower SEP than Danish-born residents [33], this may contribute to a higher risk of non-initiation or early discontinuation of AD treatment in the immigrant population.

Nevertheless, knowledge on psychiatric morbidity and psychopharmacological treatment among immigrants in Denmark is scarce. Folmann and Joergensen [34] found that first- and second-generation immigrants from several non-Western countries had lower consumption of ADs than Danish-born residents, even in minority groups who were more frequently in contact with the psychiatric system than Danish-born residents [34]; this could indicate an under-treatment in the minority groups.

The aim of the present study was to examine whether immigrants from non-Western countries are at greater risk than Danish-born residents of not receiving pharmacological therapy according to guidelines after hospitalization for moderate to severe depression. We focused on two research questions:

1) Are immigrants at greater risk than Danish-born residents of not initiating AD treatment after discharge?

2) Among those initiating therapy, are immigrants at greater risk for early AD discontinuation than Danish-born residents?

## Methods

### Study population and design

We performed a cohort study based on register-data on immigrants and Danish-born reference residents. Immigrants were defined as persons born abroad who had acquired Danish residence permission as refugees or through family reunification. Work and student migrants were not included. Furthermore, we did not include immigrants born in Western countries (defined as the EU countries, Andorra, Iceland, Lichtenstein, Monaco, Norway, San Marino, Switzerland, the Vatican, Canada, the United States, Australia, and New Zealand), assuming that these immigrants due to shared ethnic, cultural, political and socioeconomic characteristics may experience fewer barriers when faced with the Danish health care system, and may resemble Danish-born residents more in medication-taking behavior than immigrants from other countries. Danish-born residents were defined as persons born in Denmark by Danish-born parents.

The immigrant population was established using data from the Statistical Department at the Danish Immigration Service and included all refugees and family reunified individuals aged >17 years who received residence permits between 1 January 1993 and 31 December 1999. Using data from the Demographic Database at Statistics Denmark, a Danish-born reference cohort was established corresponding to four age- and sex-matched Danish-born residents per immigrant. In total, the original cohort consisted of 312,300 individuals. For details see Norredam et al. [35,36].

By means of encrypted civil registration numbers, the cohort was followed in the nation-wide Danish registries, including the Danish Psychiatric Central Register [37], the Danish National Patient Register [38], the Danish National Prescription Registry (DNPR) [39], and the socio-demographic registries (date of death, migration, and socio-economic information) [40].

The Danish Psychiatric Central Register [37] contains data on all admissions to Danish psychiatric in-patient facilities since 1969. For the purpose of this study, we selected cohort members who had been admitted with moderate to severe single episode or recurrent depression ICD-10 diagnoses F32-F33, excluding patients with mild depression (F32.0, F32.00, F32.01, F33.0, F33.00, and F33.01) and patients currently in remission (F33.4). We selected only Danish-born residents and immigrants with longer-term admissions (at least 3 days) between 1 January 1996 and 31 May 2008, corresponding to 1,808 individuals. The first admission satisfying these criteria for each patient will hereafter be referred to as the *index admission*. Information on AD dispensing was followed in the DNPR containing individual-level information on dispensed prescriptions from Danish outpatient pharmacies [39]. ADs were defined as pharmaceuticals in Anatomical Therapeutical Chemical (ATC) class N06A [41].

To explore whether immigrants and Danish-born residents were equally likely to receive the recommended AD treatment after discharge from psychiatric hospitalization, we followed the cohort in the registries until: AD dispensing, hospitalization, death, or emigration. Treatment initiation and early discontinuation were applied as outcome measures.

### Exclusion criteria

**Figure 1:**
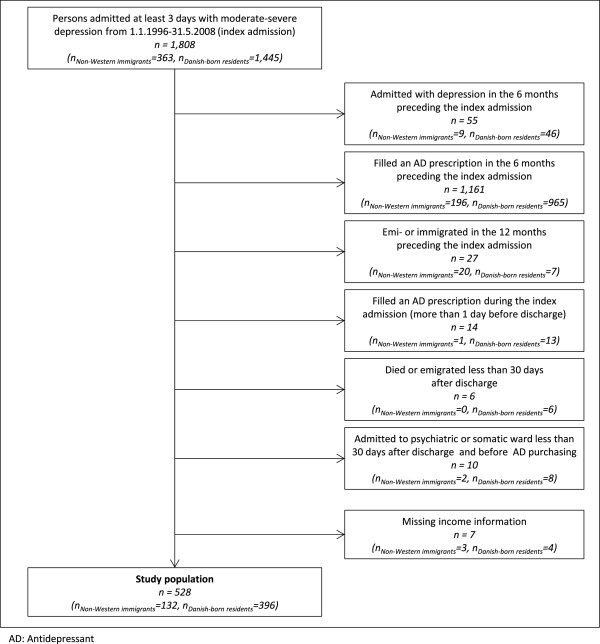
Flowchart of the population included as study population.

In order to restrict the study population to patients with incident episodes of depression, we excluded patients previously hospitalized with depression (n = 55) and patients with AD dispensing (n = 1,161) within six months prior to the index admission, Figure 1. To ensure full register information one year before the index admission, we also excluded individuals with less than one year’s residence permission or migration one year before the index admission (n = 27). Furthermore, we excluded individuals who filled prescriptions during the index admission more than one day before discharge (n = 14), and patients who died or emigrated within the first 30 days after discharge (n = 6). Finally, since the DNPR does not contain information on drugs administered under hospitalization, we excluded patients with readmissions to any hospital (psychiatric or otherwise) during the first 30 days after discharge and before purchase of ADs (n = 10), resulting in a study population of 528 individuals (132 immigrants and 396 Danish-born residents), Figure 1.

### Outcomes

*Treatment initiation*: Patients were considered to have initiated treatment if they had filled a prescription for ADs within 30 days after discharge (index prescription). Prescriptions filled the day before discharge were included, assuming that getting the required medication at the pharmacy just before discharge is part of a smooth discharge from psychiatric hospitalization (10 patients filled a prescription one day before discharge).

*Early treatment discontinuation* was explored in the cohort of patients initiating AD treatment within 30 days after discharge (n = 378), and defined as discontinuation within 180 days after the index prescription. Two methods were applied to assess early discontinuation: the minimum refill method [42] and the maximum gap method [43,44]. in line with Hansen and colleagues [19], patients were considered to have discontinued early if no further AD dispensing was observed within 180 days after the first dispensing. In this analysis, we excluded individuals who died (n = 1), emigrated (n = 1), or were hospitalized (n = 64) within 180 days after discharge, without prior filling of a second AD prescription. This resulted in a minimum refill study population of 312 individuals.

According to the maximum gap method [44], treatment is considered as discontinued if gaps in drug supply exceeds a predefined time period (grace period). In the present study, the discontinuation date among individuals having initiated therapy after discharge (n = 378) was defined as the first date when the gap between two consecutive dispensings exceeded the number of tablets in the previous dispensing plus a 50% grace period. Sensitivity analyses were performed using grace periods of 33% and 70% of number of tablets on the previous prescription. Since the DNPR does not contain information on prescribed daily dose [39], we assumed in line with Rosholm and colleagues [45] that a prescribed daily dose of AD therapy corresponds to one tablet per day. If more than one AD category or two packets of AD of the same category with different strength were dispensed the same day, we based our calculations on the largest pack size.

### Confounders and mediators

*Sex* and *age* (at the time of index admission) were included as confounders. All other co-variables were viewed as potential mediators of an association between patient origin and treatment initiation/discontinuation.

*Patient income* was used as an indicator of SEP. From the registers we retrieved information on annual personal income (including all earnings, capital income, and social transfers) in the year before the index admission.

From the Psychiatric Central Register, we obtained information on *depression severity* (moderate (ICD-10 codes F32.1, F33.1), severe (F32.2-F32.3, F33.2-F33.3), or other/unspecified (F32.8-F32.9, F33.8-F33.9)), and *type of care* to which patients were referred after discharge (coded as general practitioner, specialized treatment, no medical follow-up, or other/unknown). For patients who initiated antidepressant treatment, we included information from the DNPR on *type of antidepressant* prescribed on the first prescription after discharge tricyclic antidepressants (TCAs, ATC-class N06AA) or newer ADs (ATC-class N06AB, N06AX).

### Statistical analysis

Preliminary analyses of bivariate associations were performed using Fisher’s exact test for binary/nominal variables and gamma-test for ordinal variables. Logistic regression models were applied to assess whether post-hospitalization AD treatment was initiated independently of patient-origin, estimating odds ratio (OR) for not initiating AD treatment within 30 days after discharge for immigrants versus Danish-born residents (n = 528). Analogously, logistic regression models were applied to test early discontinuation according to the minimum refill method, estimating OR for not filling a second AD prescription within 180 days after the first dispensing (n = 312). Model calibration was performed using Hosmer and Lemeshow’s goodness-of-fit test.

Cox proportional hazard regression analyses were applied to assess whether early discontinuation was independent of patient origin, estimating hazard ratio (HR) for discontinuation defined according to the maximum gap method. Number of days since first AD dispensing was used as the underlying time variable with a follow-up time up to 180 days. Participants were censored at date of death, emigration (n = 1) or hospital admission (n = 114) before the end of follow-up. The proportional hazard assumption was tested by including the term [log(time)*patient origin] in the model. There was no evidence of violation of the proportionality assumption.

Applying a 95% confidence interval (CI), we first examined the effect of patient origin on treatment initiation/discontinuation, controlling for confounders (age and sex). Subsequently, we included potential intermediate variables in the regression models to examine their mediating effect [46]. Income was included as a categorical variable, divided into four groups: <100,000 Danish Krone (DKK); 100,000-199,999 DKK; 200,000-299,999 DKK; and ≥300,000 DKK (100 EUR = 746 DKK; November 2012). Age was included as a continuous variable. Use of alternative specifications did not alter conclusions.

All statistical analyses were carried out in SAS, version 9.2. Register-based studies in Denmark do not require approval by an ethics board, but the study was approved by the Danish National Data Protection Agency.

## Results

### Description of study population

Table 1 presents the main characteristics of the study population of patients discharged after hospitalization with depression (n = 528) along with descriptive data on post-hospitalization AD treatment. For both immigrants and Danish-born residents, the majority of the population was younger (18–39 years) and female. A higher proportion of immigrants than Danish-born residents were in lower income groups (p < 0.001). There were no significant differences between immigrants and Danish-born residents regarding depression severity, type of AD on first prescription, or type of care after discharge. Therefore, income was the only covariate included as a potential mediator in subsequent multivariate analyses [46].

**Table 1:** Characteristics of study population (n = 528)

**Population characteristics**	**Danish-born residents n = 396 (%)**	**Non-Western immigrants n = 132 (%)**	**P**^**1**^
**Sex**			
Female	232 (58.6%)	78 (59.1%)	0.92
**Age at index admission (years)**			
18-29	98 (24.8%)	17 (12.9%)	0.10
30-39	158 (39.9%)	65 (49.2%)	
40-49	87 (22.0%)	33 (25.0%)	
50-59	26 (6.6%)	11 (8.3%)	
60-86	27 (6.8%)	61 (4.6%)	
**Annual income**^**2**^			
<13,405 EUR	52 (13.1%)	31 (23.5%)	<0.0001
13,405-26,809 EUR	182 (46.0%)	76 (57.6%)	
26,810-40,214 EUR	120 (30.3%)	21 (15.9%)	
≥40,215 EUR	42 (10.6%)	4(3.0%)	
**Severity of depression at admission**			
Moderate	185 (46.7%)	51 (38.6%)	0.27
Severe	179 (45.2%)	69 (52.3%)	
Other type/unspecified	32 (8.1%)	12 (9.1%)	
**Type of outpatient care referred to after discharge**			
General practitioner	142 (35.9%)	54 (40.9%)	0.46
Specialized treatment	240 (60.6%)	75 (56.8%)	
No medical follow-up/Other/unknown	14 (3.5%)	3 (2.3%)	
**Post-discharge AD treatment**			
No treatment initiation within 30 days	104 (26.3%)	46 (34.8%)	0.07
Early discontinuation (minimum refill, n = 312)	28 (11.6%)	13 (18.3%)	0.16
**Type of AD on first prescription after discharge**			
TCA^3^	43 (14.7%)	12 (14.0%)	1.00
Newer ADs^4^	249 (85.3%)	74 (86.1%)	

*AD* Antidepressant, *EUR* Euro, *TCA* Tricyclic antidepressant.

^1^Two-sided Fisher’s exact test (two-sided gamma-test for ordinal variables).

^2^100 EUR = 746 Danish Krone (DKK) (November 2012).

^3^ATC codes: N06AA04, N06AA09-10, N06AA12, N06AA16.

^4^ATC codes: N06AB03, N06AB04-10, N06AX03, N06AX11, N06AX16, N06AX21.

Table 2 presents the distribution of immigrants (n = 132) by their region of origin. The immigrants mainly originated in the Former Yugoslavia (n = 60, 45.5%) and the Middle East (n = 46, 34.9%). When looking at the specific country of origin, 37.7% (n = 50) of the immigrants were born in Bosnia-Herzegovina, 16.7% (n = 22) in Iraq, 7.6% (n = 10) in Iran, 4.5% (n = 6) in Turkey, and 3.8% (n = 5) in Somalia.

**Table 2:** Distribution of non-Western immigrants by region of origin (n = 132)

**Region**	**N (%)**
Eastern Europe (excl. Former Yugoslavia)	5 (3.8%)
Former Yugoslavia	
Bosnia-Herzegovina	50 (37.9%)
Other Former Yugoslavia	10 (7.6%)
Middle East	
Turkey	6 (4.5%)
Iran	10 (7.6%)
Iraq	22 (16.7%)
Other Middle East	8 (6.1%)
Africa	
Somalia	5 (3.8%)
Other African countries	9 (6.8%)
South- and Middle America	2 (1.5%)
Asia	5 (3.8%)

### Treatment initiation

A higher proportion of immigrants (34.8%) than Danish-born patients (26.3%) did not initiate AD treatment within 30 days after discharge (p = 0.07), Table 1. Table 3 shows the multivariate analyses of not initiating AD treatment. When adjusting for gender and age, immigrants had higher odds for not initiating AD treatment than Danish-born residents (OR = 1.55; 95% CI: 1.01-2.38). When income was included as a potential mediator, the strength of the association between patient origin and treatment initiation was attenuated (1.31; 0.84-2.03) and was not statistically significant. Assuming the same gender pattern across the two ethnic groups, the analysis also showed that men had significantly higher odds than women for not initiating AD treatment (1.59; 1.08-2.34), Table 3.

**Table 3:** Odds Ratio (OR) for not initiating AD treatment within 30 days after discharge Non-Western immigrants are compared with Danish-born residents (n = 528, events = 150)

	**Model 1**	**Model 2**
**Patient characteristic**	**OR (95% CI)**	**OR (95% CI)**
**Patient origin**		
Danish-born residents (ref)	1	1
Non-Western immigrants	1.55 (1.01-2.38)	1.31 (0.84-2.03)
**Sex**		
Female (ref)	1	1
Male	1.59 (1.08-2.34)	1.71 (1.15-2.53)
**Age**		
Continuous	0.98 (0.96-1.00)	0.98 (0.97-1.00)
**Annual income**^**1**^		
<13,405 EUR (ref)		1
13,405-26,809 EUR		0.98 (0.58-1.66)
26,810-40,214 EUR		0.48 (0.25-0.89)
≥40,215 EUR		0.29 (0.11-0.79)

*AD* antidepressant treatment, *CI* Confidence interval, *EUR* Euro, *OR* Odds ratio.

Model 1: OR for not initiating treatment, including patient origin, age and sex.

Model 2: OR for not initiating treatment, including patient origin, age, sex and income.

^1^100 EUR = 746 Danish Krone (DKK) (November 2012).

### Early treatment discontinuation

A higher proportion of immigrants (18.3%) than Danish-born residents (11.6%) discontinued AD treatment early according to the minimum refill method, but this difference was not statistically significant, p = 0.16 (Table 1). Table 4 presents the multivariate analyses of early discontinuation defined according to the minimum refill method and the maximum gap method, respectively. Adjusting for age and gender in the minimum refill approach (model A1), odds for early discontinuation was insignificantly higher among immigrants than Danish-born residents (OR = 1.80; 95% CI: 0.87-3.73). When income was included in the model, the strength of the association between patient origin and early treatment discontinuation was only slightly attenuated, and income was not itself a significant predictor of early discontinuation (model A2).

**Table 4:** Early discontinuation of AD treatment within 180 days after discharge, applying A) The minimum refill method and B) The maximum gap method (50% supply gap)

	**A. Minimum refill method**				**B. Maximum gap method**			
	**OR (95% CI)**				**HR (95% CI)**			
	**n = 312;events = 41**				**n = 378;events = 72**			
**Patient characteristic**	**Model A1**		**Model A2**		**Model B1**		**Model B2**	
**Patient origin**								
Danish-born residents (ref)	1		1		1		1	
Non-Western immigrants	1.80 (0.87-3.73)	(p = 0.12)	1.69 (0.79-3.59)	(p = 0.17)	1.46 (0.87-2.45)	(p = 0.15)	1.52 (0.89-2.61)	(p = 0.13)
**Sex**								
Female (ref)	1		1		1		1	
Male	1.82 (0.93-3.55)	(p = 0.08)	1.90 (0.96-3.76)	(p = 0.06)	1.58 (0.99-2.52)	(p = 0.05)	1.59 (0.99-2.55)	(p = 0.05)
**Age**								
Continuous	0.98 (0.95-1.01)	(p = 0.23)	0.98 (0.95-1.01)	(p = 0.19)	0.99 (0.97-1.01)	(p = 0.17)	0.98 (0.96-1.00)	(p = 0.12)
**Annual income**^**1**^								
<13,405 EUR (ref)			1				1	
13,405-26,809 EUR			0.73 (0.29-1.81)	(p = 0.93)			0.75 (0.40-1.40)	(p = 0.36)
26,810-40,214 EUR			0.43 (0.15-1.28)	(p = 0.13)			0.57 (0.28-1.18)	(p = 0.13)
≥40,215 EUR			0.81 (0.23-2.87)	(p = 0.75)			1.24 (0.53-2.87)	(p = 0.62)

*AD* Antidepressant, *EUR* Euro, *HR* Hazard ratio, *OR* Odds ratio.

Model A1: OR for not refilling a prescription within 180 days after the first prescription, including patient origin, age and sex.

Model A2: OR for not refilling a prescription within 180 days after the first prescription, including patient origin, age, sex and income.

Model B1: HR for not refilling a prescription within the allowed supply gap, including patient origin, age and sex.

Model B2: HR for not refilling a prescription within the allowed supply gap, including patient origin, age, sex and income.

^1^100 EURO = 746 Danish Krone (DKK) (November 2012).

Analyses based on the maximum gap method revealed similar results. Applying a supply gap of maximum 50% (model B1), immigrants were more likely to discontinue AD therapy prematurely than Danish-born residents, but this difference did not reach statistical significance (HR = 1.46; 95% CI: 0.87-2.45). Including income only marginally changed the strength of the association (model B2). Sensitivity analyses using maximum supply gaps of 30% and 70% indicated that point estimates were relatively robust to the gap definition, although the association between patient origin and early discontinuation was attenuated when only allowing a supply gap of 30% (HR=1.27; 95% CI: 0.77-2.10).

## Discussion

### Main findings

In this register-based cohort study, a lower proportion of non-Western immigrants than Danish-born residents initiated AD treatment within 30 days after discharge from hospitalization with depression (34.8% versus 26.3%). Among those who initiated therapy, a higher proportion of the immigrants discontinued treatment within 180 days (18.3% versus 11.6%).

Immigrants were significantly less likely than Danish-born residents to initiate AD treatment after discharge. When including income in the model, the difference in treatment initiation was attenuated, indicating that income partly mediated the observed difference. Applying two different methods for measuring treatment discontinuation, both analyses showed that immigrants were more likely to discontinue AD treatment early than Danish-born patients, but the observed trends were not statistically significant. Income did not seem to mediate the trend toward ethnic disparities in premature discontinuation.

### Initiation and early discontinuation of AD treatment

Our findings are in accordance with previous studies indicating that minority groups are at higher risk than majority populations of not receiving the recommended AD treatment for depression [9,10,19,21,24-26,47,48]. However, to our knowledge no prior studies have restricted the study population to patients discharged from hospitalization with moderate to severe depression and explored both initiation of AD treatment after hospitalization and early discontinuation of therapy. In line with our study, a register-based study from the US [25] showed that minorities (African Americans, Asians, and Hispanics) with newly diagnosed depression were less likely to receive adequate acute- and post-remission antidepressant treatment than the majority population. A survey-based US study on outpatients admitted to mental health clinics with major depression [24] showed that the majority population (white Americans) were almost three times more likely to be recommended AD treatment than the minority population. In a recent register-based cohort study from Spain, Cruz and colleagues found that immigrant patients were more likely than native patients to discontinue AD treatment during follow-up, and nearly half of the immigrant patients discontinued treatment within the first month [26]. Although studies in this field are difficult to compare due to different health care- and reimbursement systems, different types of ethnic minorities, different clinical settings, and different approaches for measuring AD initiation and discontinuation, the studies all indicate that minority groups are less likely to receive adequate pharmacological therapy for depression than the majority population [9,10,19,21,24-26,47,48].

Adding to previous findings from other countries, our Danish study indicates that even in a predominantly tax-financed health care system, ethnic disparities appear to exist as to initiation of AD treatment when comparing first-generation immigrants with Danish-born patients. As the physician responsible for psychiatric discharge often also is responsible for planning out-patient treatment, the first AD prescription is most likely prescribed by the hospital psychiatrist. Thus, the high proportion of patients (independent of origin) not initiating therapy could indicate that psychiatric wards are confronted with challenges of preparing patients better for the post-hospitalization pharmacological therapy. The fact that immigrants have lower odds for initiating therapy could indicate that cultural- or migration-related factors are at play (see Potential Mechanisms below).

To our knowledge no Danish studies have previously explored ethnic differences in initiation of AD treatment after psychiatric hospitalization. Applying the same minimum refill definition of early AD discontinuation as in our study, Hansen and colleagues [19] use Danish/foreign citizenship rather than country of birth as a measure of patient origin in the aforementioned Danish study on predictors for early AD discontinuation. In contrast to our study, the study by Hansen and colleagues includes all patients initiating AD treatment in general practice, i.e. also when AD treatment is initiated for indications other than moderate to severe depression, such as mild depression or anxiety disorders. As a consequence, their study population is very large compared to our study, which may partly explain their finding of a significantly higher odds for early discontinuation among foreign citizens compared with Danish citizens (OR = 1.99, 99% CI: 1.18-3.37), whereas the association in our study was non-significant (OR = 1.80, 95% CI: 0.87-3.73).

### Potential mechanisms for ethnic disparities in AD treatment

To improve mental health care for immigrant populations, it is important to uncover factors explaining ethnic disparities in the recommended post-hospitalization AD treatment. We explored to what extent income mediated the differences in AD treatment according to patient origin. In line with two previous Danish studies on ethnic disparities in pharmacological therapy for cardiovascular disease [8] and diabetes [49], we found that income only to a limited extent mediated the observed ethnic disparities. While some studies have shown that adherence to pharmacological therapy increases with income [19,21], other studies have found that adherence decreases with increasing SEP [50]. It may be speculated that individuals with high SEP could be less likely to accept the side effects of AD treatment, since they have the possibility to pay for costly alternatives such as psychotherapy. Thus, the relationship between SEP and adherence is not straightforward, which may explain the limited mediating effect of this variable. Our analysis also indicated that the ethnic disparities in post-hospitalization AD treatment were not explained by depression severity, type of AD treatment (newer ADs versus TCA), or type of out-patient care after discharge.

Hence, factors which could not be thoroughly examined in the present register-based study seem to be at play as to the ethnic disparities in post-hospitalization AD therapy. Such potential factors may include patient beliefs and social norms regarding AD treatment [30-32]; health care providers’ attitudes towards immigrant patients [12]; and the cross-cultural communication skills of health care providers [11]. Moreover, ethnic differences in the acceptability of AD treatment [27-29], differences in the beliefs about the causes of and potential cures for illness [51,52], or differences in the social stigma associated with depression and AD treatment [53] may influence patient medication-taking behavior [30-32]. Furthermore, stressors related to migration [54,55] may be hypothesized to influence immigrant patients’ mental resources to cope with disease and adhere to treatment. As regards the health care provider, the ability to establish a patient-provider relationship characterized by clear communication, trust and mutual understanding is essential to ensuring patient adherence [18,20,54,56,57]. Several studies have found that meetings between health care professionals and immigrant patients may sometimes be characterized by miscommunication, misunderstandings and frustration [11,12,58,59]. In this regard, access to professional interpreter assistance is likely to be an important organizational strategy to ensure quality of care for immigrant patients [60]. Furthermore, poor collaboration and coordination between primary and secondary health care sector may increase the risk of treatment discontinuation after discharge from psychiatric hospital. This may especially be the case for immigrant patients, who may lack knowledge of the structure and functioning of the Danish health care system, since immigrants are not systematically introduced to the health care system [61].

In a previous study [36], we demonstrated that immigrants are more frequently subjected to coercive measures during psychiatric hospitalization than native Danes, potentially due to delayed admission to psychiatric wards or to cultural barriers and misunderstandings [58]. Hence, the observed excess in coercive measures applied to ethnic minorities [36] may introduce mistrust and skepticism towards the pharmacological treatment recommended by health care professionals. Unfortunately, we did not have access to information regarding coercive measures for the entire observation period in the present study. Thus, further research is needed to uncover to what extent use of coercive measures in psychiatry increases the risk of non-adherence to post-hospitalization pharmacological therapy.

### Strengths and limitations of the study

The main strength of our study is the linkage of individual-level data from various Danish registers. This allowed us to establish a homogenous cohort of individuals hospitalized with moderate-severe depression that could be followed for subsequent AD dispensing and other register information without the risk of selection or information bias.

However, we also acknowledge several limitations of our study. First of all, due to our small sample size, we were unable to conduct analyses according to the immigrants’ specific country of origin. Immigrants from non-Western countries are a heterogeneous group with a variety of social, linguistic, ethnic and religious backgrounds, and medication-taking behavior may differ between immigrants of different origin [8,34]. Therefore, it may be considered simplistic to regard immigrants as one group. Even an immigrant’s specific country of origin may be a very crude proxy for ethnicity [62,63]. It has been argued that self-reported ethnicity is a more precise, dynamic and non-discriminatory measure [62,63]. However, self-reported ethnicity was not available in the present register-based study.

Secondly, our relatively small sample size also meant that we had few discontinuation events. This increased the risk of not finding statistically significant differences in AD treatment according to patient origin and resulted in wide 95% confidence intervals in the multivariate models. Lack of statistical power is a general problem when conducting epidemiological studies on immigrants, not least in countries like Denmark with relatively small immigrant populations – especially when the immigrant population is limited to individuals acquiring residence permits as refugees or by family reunification [36].

We opted to examine the robustness of our results by using two different approaches for evaluating early discontinuation and by conducting sensitivity analyses, which allowed varying lengths of gaps in medication supply. Applying data from DNPR on dispensed prescription drugs as a proxy for whether patients receive the recommended pharmacological treatment rests upon several assumptions [39]: 1) patients consume the redeemed medicine (*which we assume here*), and 2) any missing redemption is caused either by the physician (no prescription prescribed during the meeting with the patient) or by the patient failing to fill the prescription. Both aspects of missing drug redemption are of interest, but in this study we could not distinguish between the two.

Thirdly, we applied personal income as the only indicator for SEP. Applying educational level as an SEP indicator could have provided the study with supplementary aspects, since different indicators of SEP may represent different mechanisms through which SEP impacts on treatment initiation and discontinuation [64]. While an association between income and educational level exists in native born populations, the same association may not exist in immigrant populations due to difficulties in obtaining jobs related to obtained education. However, we were not able to include this variable in the analyses as the educational level of immigrants is incompletely recorded in the Danish registers. The limitations of applying personal income as an indicator for economic capacity should also be recognized, especially when comparing ethnic groups with different family structures. In our analysis, the lowest income group tended to have less economic barriers for buying medicines than the medium income groups. This paradox may be explained by the fact that personal income does not include special social transfers such as rent subsidy and supplementary drug reimbursement (requiring application). If Danish-born residents are more likely than immigrants to receive supplementary reimbursement for out-of-pocket drug expenditures, we may have underestimated income related disparities.

Fourthly, our study includes only information on medicine purchased in Denmark. However, a recent Danish study [65] showed that immigrants in Denmark tend to buy medicines abroad, potentially explaining part of the observed disparities as regards to AD prescription dispensed at Danish pharmacies.

Lastly, since our study included refugees and family reunited immigrants only, our findings may not be generalizable to other immigrant groups such as work migrants.

### Implications

Although our small sample size warrants caution when drawing conclusions, the results of our study indicate a need for interventions to improve communication between health care professionals and immigrant patients. An important element in this may be to ensure professional interpreter assistance for immigrant patients with limited proficiency in the local language [60]. Nevertheless, in 2011, user-fees (now abolished) were introduced for interpreter assistance to immigrants with permanent residency in Denmark. It has recently been shown that these user fees resulted in less use of interpreters, especially in lower income groups [66]. A close and genuine patient/provider dialogue about the planned treatment after hospitalization is likely to be crucial for ensuring AD adherence after discharge [18,57]. Furthermore, our results point to a need for efforts to improve collaboration between primary and secondary health care providers in order to ensure treatment continuity after hospital discharge [67], e.g. by improved communication about patients’ discharge plans between inpatient staff and outpatient clinicians [68].

## Conclusion

Compared to Danish-born residents, refugees and family reunited immigrants from non-Western countries seem to be less likely to receive the recommended AD treatment after hospitalization with moderate to severe depression. This is especially true for the first AD dispensing after discharge, indicating special communication problems when negotiating pharmacological therapy with immigrant patients under hospitalization, along with a need for improving collaboration between secondary and primary care to ensure continuity in treatment. Albeit not statistically significant, we also observed a tendency towards ethnic disparities in early AD treatment discontinuation. Only a small part of the ethnic disparities were explained by income differences.

## Competing interest

The authors declare that they have no competing interest.

## Authors’ contributions

LTT, MLN, JHP, and HWK designed the study, which formed the basis of LTT’s master’s thesis. HWK designed a re-analysis of the data and wrote the manuscript. MK performed the analyses. MLN provided information for the database in Statistics Denmark. All authors reviewed the manuscript and contributed with comments. All authors read and approved the final manuscript.

## Pre-publication history

The pre-publication history for this paper can be accessed here:

http://www.biomedcentral.com/1471-244X/14/77/prepub

## References

[B1] Statistics Denmark STATBANK Statistics Denmark. http://www.statistikbanken.dk/statbank5a/default.asp?w=1280 Accesed 22-3-2012

[B2] Tinghog P Hemmingsson T Lundberg I To what extent may the association between immigrant status and mental illness be explained by socioeconomic factors? Soc Psychiatry Psychiatr Epidemiol 2007 42 12 990 996 17846697 10.1007/s00127-007-0253-5

[B3] van der Wurff FB Beekman AT Dijkshoorn H Spijker JA Smits CH Stek ML Verhoeff A Prevalence and risk-factors for depression in elderly Turkish and Moroccan migrants in the Netherlands J Affect Disord 2004 83 1 33 41 15546643 10.1016/j.jad.2004.04.009

[B4] Syed HR Dalgard OS Hussain A Dalen I Claussen B Ahlberg NL Inequalities in health: a comparative study between ethnic Norwegians and Pakistanis in Oslo, Norway Int J Equity Health 2006 5 7 16808838 10.1186/1475-9276-5-7 PMC1553452

[B5] Bhugra D Migration and depression Acta Psychiatr Scand Suppl 2003 418 67 72 12956818 10.1034/j.1600-0447.108.s418.14.x

[B6] Olejaz M Juul NA Rudkjobing A Okkels BH Krasnik A Hernandez-Quevedo C Denmark health system review Health Syst Transit 2012 14 2 i 192 22575801

[B7] Pedersen KM Pricing and reimbursement of drugs in Denmark Eur J Health Econom 2003 4 60 65 10.1007/s10198-003-0165-6 15609170

[B8] Hempler NF Diderichsen F Larsen FB Ladelund S Jorgensen T Do immigrants from Turkey, Pakistan and Yugoslavia receive adequate medical treatment with beta-blockers and statins after acute myocardial infarction compared with Danish-born residents? A register-based follow-up study Eur J Clin Pharmacol 2010 66 7 735 742 20393695 10.1007/s00228-010-0816-3

[B9] van Dijk L Heerdink ER Somai D van Dulmen S Sluijs EM de Ridder DT Griens AM Bensing JM Patient risk profiles and practice variation in nonadherence to antidepressants, antihypertensives and oral hypoglycemics BMC Health Serv Res 2007 7 51 17425792 10.1186/1472-6963-7-51 PMC1855317

[B10] Charbonneau A Rosen AK Ash AS Owen RR Kader B Spiro A III Hankin C Herz LR Jo VP Kazis L Miller DR Berlowitz DR Measuring the quality of depression care in a large integrated health system Med Care 2003 41 5 669 680 12719691 10.1097/01.MLR.0000062920.51692.B4

[B11] Schouten BC Meeuwesen L Cultural differences in medical communication: a review of the literature Patient Educ Couns 2006 64 1–3 21 34 16427760 10.1016/j.pec.2005.11.014

[B12] Michaelsen J Krasnik A Nielsen A Norredam M Torres AM Health professionals’ knowledge, attitudes, and experiences in relation to immigrant patients: a questionnaire study at a Danish hospital Scand J Public Health 2004 32 4 287 295 15370769 10.1080/14034940310022223

[B13] Norredam M Migrants’ access to healthcare Dan Med Bull 2011 58 10 B4339 21975158

[B14] Anderson IM Ferrier IN Baldwin RC Cowen PJ Howard L Lewis G Matthews K Lister-Williams RH Peveler RC Scott J Tylee A Evidence-based guidelines for treating depressive disorders with antidepressants: a revision of the 2000 British Association for Psychopharmacology guidelines J Psychopharmacol 2008 22 4 343 396 18413657 10.1177/0269881107088441

[B15] American Psychiatric Association Practice guideline for the treatment of patients with major depressive disorder (revision) Am J Psychiatry 2000 157 4 Suppl 1 45 10767867

[B16] National Institute for Health and Clinical Excellence Depression in Adults (Update): The Treatment and Management of Depression in Adults. National Clinical Practice Guideline 90 2009 London: The British Psychological Society and The Royal College

[B17] Danish National Board of Health Reference Program for Unipolar Depression hos voksne [Reference Program for Treatment of Unipolar Depression in Adults] 2007 Copenhagen: Danish National Board of Health

[B18] Bull SA Hu XH Hunkeler EM Lee JY Ming EE Markson LE Fireman B Discontinuation of use and switching of antidepressants: influence of patient-physician communication JAMA 2002 288 11 1403 1409 12234237 10.1001/jama.288.11.1403

[B19] Hansen DG Vach W Rosholm JU Sondergaard J Gram LF Kragstrup J Early discontinuation of antidepressants in general practice: association with patient and prescriber characteristics Fam Pract 2004 21 6 623 629 15520034 10.1093/fampra/cmh608

[B20] Lin EH Von KM Katon W Bush T Simon GE Walker E Robinson P The role of the primary care physician in patients’ adherence to antidepressant therapy Med Care 1995 33 1 67 74 7823648 10.1097/00005650-199501000-00006

[B21] Olfson M Marcus SC Tedeschi M Wan GJ Continuity of antidepressant treatment for adults with depression in the United States Am J Psychiatry 2006 163 1 101 108 16390896 10.1176/appi.ajp.163.1.101

[B22] Lingam R Scott J Treatment non-adherence in affective disorders Acta Psychiatr Scand 2002 105 3 164 172 11939969 10.1034/j.1600-0447.2002.1r084.x

[B23] van Geffen EC Gardarsdottir H Van HR Van DL Egberts AC Heerdink ER Initiation of antidepressant therapy: do patients follow the GP’s prescription? Br J Gen Pract 2009 59 559 81 87 19192372 10.3399/bjgp09X395067 PMC2629822

[B24] Sirey JA Meyers BS Bruce ML Alexopoulos GS Perlick DA Raue P Predictors of antidepressant prescription and early use among depressed outpatients Am J Psychiatry 1999 156 5 690 696 10327900 10.1176/ajp.156.5.690

[B25] Virnig B Huang Z Lurie N Musgrave D McBean AM Dowd B Does Medicare managed care provide equal treatment for mental illness across races? Arch Gen Psychiatry 2004 61 2 201 205 14757597 10.1001/archpsyc.61.2.201

[B26] Cruz I Serna C Rue M Real J Soler-Gonzalez J Galvan L Duration and compliance with antidepressant treatment in immigrant and native-born populations in Spain: a four year follow-up descriptive study BMC Public Health 2012 12 256 22469197 10.1186/1471-2458-12-256 PMC3350418

[B27] Thorens G Gex-Fabry M Zullino DF Eytan A Attitudes toward psychopharmacology among hospitalized patients from diverse ethno-cultural backgrounds BMC Psychiatry 2008 8 55 18613960 10.1186/1471-244X-8-55 PMC2478676

[B28] Wagner AW Bystritsky A Russo JE Craske MG Sherbourne CD Stein MB Roy-Byrne PP Beliefs about psychotropic medication and psychotherapy among primary care patients with anxiety disorders Depress Anxiety 2005 21 3 99 105 15965996 10.1002/da.20067

[B29] Cooper LA Gonzales JJ Gallo JJ Rost KM Meredith LS Rubenstein LV Wang NY Ford DE The acceptability of treatment for depression among African-American, Hispanic, and white primary care patients Med Care 2003 41 4 479 489 12665712 10.1097/01.MLR.0000053228.58042.E4

[B30] Aikens JE Nease DE Jr Nau DP Klinkman MS Schwenk TL Adherence to maintenance-phase antidepressant medication as a function of patient beliefs about medication Ann Fam Med 2005 3 1 23 30 15671187 10.1370/afm.238 PMC1466796

[B31] Brown C Battista DR Bruehlman R Sereika SS Thase ME Dunbar-Jacob J Beliefs about antidepressant medications in primary care patients: relationship to self-reported adherence Med Care 2005 43 12 1203 1207 16299431 10.1097/01.mlr.0000185733.30697.f6

[B32] Hunot VM Horne R Leese MN Churchill RC A cohort study of adherence to antidepressants in primary care: the influence of antidepressant concerns and treatment preferences Prim Care Companion J Clin Psychiatry 2007 9 2 91 99 17607330 10.4088/pcc.v09n0202 PMC1896312

[B33] Statistics Denmark Indvandrere i Danmark 2008 [Immigrants in Denmark 2008] 2008 Copenhagen: Statistics Denmark

[B34] Folmann NB Jørgensen T Etniske minoriteter - sygdom og brug af sundhedsvæsenet. Et registerstudie [Ethnic minorities - illness and use of the health care system. A register-based study] 2006 Copenhagen: Danish National Board of Health

[B35] Norredam M Garcia-Lopez A Keiding N Krasnik A Risk of mental disorders in refugees and native Danes: a register-based retrospective cohort study Soc Psychiatry Psychiatr Epidemiol 2009 44 12 1023 1029 19294322 10.1007/s00127-009-0024-6

[B36] Norredam M Garcia-Lopez A Keiding N Krasnik A Excess use of coercive measures in psychiatry among migrants compared with native Danes Acta Psychiatr Scand 2010 121 2 143 151 19594483 10.1111/j.1600-0447.2009.01418.x

[B37] Mors O Perto GP Mortensen PB The Danish psychiatric central research register Scand J Public Health 2011 39 7 Suppl 54 57 21775352 10.1177/1403494810395825

[B38] Lynge E Sandegaard JL Rebolj M The Danish national patient register Scand J Public Health 2011 39 7 Suppl 30 33 21775347 10.1177/1403494811401482

[B39] Kildemoes HW Sorensen HT Hallas J The Danish national prescription registry Scand J Public Health 2011 39 7 Suppl 38 41 21775349 10.1177/1403494810394717

[B40] Thygesen LC Daasnes C Thaulow I Bronnum-Hansen H Introduction to Danish (nationwide) registers on health and social issues: structure, access, legislation and archiving Scand J Public Health 2011 39 Supple 7 12 16 21898916 10.1177/1403494811399956

[B41] World Health Organization Collaborating Centre for Drug Statistics Methodology ATC - structure and principles: World Health Organization, March 2011 http://www.whocc.no/atc/structure_and_principles/. (Accessed Mar 2012 22)

[B42] Caetano PA Lam JM Morgan SG Toward a standard definition and measurement of persistence with drug therapy: examples from research on statin and antihypertensive utilization Clin Ther 2006 28 9 1411 1424 17062314 10.1016/j.clinthera.2006.09.021

[B43] van Wijk BL Klungel OH Heerdink ER De Boer A Refill persistence with chronic medication assessed from a pharmacy database was influenced by method of calculation J Clin Epidemiol 2006 59 1 11 17 16360556 10.1016/j.jclinepi.2005.05.005

[B44] Lesen E Sandstrom TZ Carlsten A Jonsson AK Mardby AC Sundell KA A comparison of two methods for estimating refill adherence to statins in Sweden: the RARE project Pharmacoepidemiol Drug Saf 2011 20 10 1073 1079 21853505 10.1002/pds.2204

[B45] Rosholm JU Andersen M Gram LF Are there differences in the use of selective serotonin reuptake inhibitors and tricyclic antidepressants? A prescription database study Eur J Clin Pharmacol 2001 56 12 923 929 11317482 10.1007/s002280000234

[B46] Baron RM Kenny DA The moderator-mediator variable distinction in social psychological research: conceptual, strategic, and statistical considerations J Pers Soc Psychol 1986 51 6 1173 1182 3806354 10.1037//0022-3514.51.6.1173

[B47] Alegria M Chatterji P Wells K Cao Z Chen CN Takeuchi D Jackson J Meng XL Disparity in depression treatment among racial and ethnic minority populations in the United States Psychiatr Serv 2008 59 11 1264 1272 18971402 10.1176/appi.ps.59.11.1264 PMC2668139

[B48] Miranda J Cooper LA Disparities in care for depression among primary care patients J Gen Intern Med 2004 19 2 120 126 15009791 10.1111/j.1525-1497.2004.30272.x PMC1492138

[B49] Sanchez-Ramirez DC Krasnik A Wallach-Kildemoes H Do immigrants from Turkey, Pakistan and Ex-Yugoslavia with newly diagnosed type 2 diabetes initiate recommended statin therapy to the same extent as Danish-born residents? A nationwide register study Eur J Clin Pharmacol 2013 69 1 87 95 22648279 10.1007/s00228-012-1306-6

[B50] ten Doesschate MC Bockting CL Koeter MW Schene AH Predictors of nonadherence to continuation and maintenance antidepressant medication in patients with remitted recurrent depression J Clin Psychiatry 2009 70 1 63 69 19192463 10.4088/jcp.08m04119

[B51] Hjelm KG Bard K Nyberg P Apelqvist J Beliefs about health and diabetes in men of different ethnic origin J Adv Nurs 2005 50 1 47 59 15788065 10.1111/j.1365-2648.2004.03348.x

[B52] Hjelm K Bard K Nyberg P Apelqvist J Religious and cultural distance in beliefs about health and illness in women with diabetes mellitus of different origin living in Sweden Int J Nurs Stud 2003 40 6 627 643 12834928 10.1016/s0020-7489(03)00020-8

[B53] Givens JL Katz IR Bellamy S Holmes WC Stigma and the acceptability of depression treatments among african americans and whites J Gen Intern Med 2007 22 9 1292 1297 17610120 10.1007/s11606-007-0276-3 PMC2219769

[B54] Mirdal GM Stress and distress in migration: problems and resources of Turkish women in Denmark Int Migr Rev 1984 18 4 Special Issue 984 1003 12340241

[B55] Emami A Torres S Making sense of illness: late-in-life migration as point of departure for elderly Iranian immigrants’ explanatory models of illness J Immigr Health 2005 7 3 153 164 15900416 10.1007/s10903-005-3672-y

[B56] Frank E Kupfer DJ Siegel LR Alliance not compliance: a philosophy of outpatient care J Clin Psychiatry 1995 56 Suppl 1 11 16 7836346

[B57] Bultman DC Svarstad BL Effects of physician communication style on client medication beliefs and adherence with antidepressant treatment Patient Educ Couns 2000 40 2 173 185 10771371 10.1016/s0738-3991(99)00083-x

[B58] Jensen NK Norredam M Priebe S Krasnik A How do general practitioners experience providing care to refugees with mental health problems? A qualitative study from Denmark BMC Fam Pract 2013 14 17 23356401 10.1186/1471-2296-14-17 PMC3568406

[B59] van Wieringen JC Harmsen JA Bruijnzeels MA Intercultural communication in general practice Eur J Public Health 2002 12 1 63 68 11968523 10.1093/eurpub/12.1.63

[B60] Karliner LS Jacobs EA Chen AH Mutha S Do professional interpreters improve clinical care for patients with limited English proficiency? A systematic review of the literature Health Serv Res 2007 42 2 727 754 17362215 10.1111/j.1475-6773.2006.00629.x PMC1955368

[B61] Frederiksen HW Krasnik A Norredam M Policies and practices in the health-related reception of quota refugees in Denmark Dan Med J 2012 59 1 A4352 22239837

[B62] Senior PA Bhopal R Ethnicity as a variable in epidemiological research BMJ 1994 309 6950 327 330 8086873 10.1136/bmj.309.6950.327 PMC2540882

[B63] Kaplan JB Bennett T Use of race and ethnicity in biomedical publication JAMA 2003 289 20 2709 2716 12771118 10.1001/jama.289.20.2709

[B64] Galobardes B Shaw M Lawlor DA Lynch JW Davey SG Indicators of socioeconomic position (part 1) J Epidemiol Community Health 2006 60 1 7 12 16361448 10.1136/jech.2004.023531 PMC2465546

[B65] Nielsen SS Yazici S Petersen SG Blaakilde AL Krasnik A Use of cross-border healthcare services among ethnic Danes, Turkish immigrants and Turkish descendants in Denmark: a combined survey and registry study BMC Health Serv Res 2012 12 390 23148550 10.1186/1472-6963-12-390 PMC3536574

[B66] Harpelund L Nielsen SS Krasnik A Self-perceived need for interpreter among immigrants in Denmark Scand J Public Health 2012 40 5 457 465 22825969 10.1177/1403494812454234

[B67] Crawford MJ de Jonge E Freeman GK Weaver T Providing continuity of care for people with severe mental illness- a narrative review Soc Psychiatry Psychiatr Epidemiol 2004 39 4 265 272 15085327 10.1007/s00127-004-0732-x

[B68] Boyer CA McAlpine DD Pottick KJ Olfson M Identifying risk factors and key strategies in linkage to outpatient psychiatric care Am J Psychiatry 2000 157 10 1592 1598 11007712 10.1176/appi.ajp.157.10.1592

